# The Walking Interventions Through Texting (WalkIT) Trial: Rationale, Design, and Protocol for a Factorial Randomized Controlled Trial of Adaptive Interventions for Overweight and Obese, Inactive Adults

**DOI:** 10.2196/resprot.4856

**Published:** 2015-09-11

**Authors:** Jane C Hurley, Kevin E Hollingshead, Michael Todd, Catherine L Jarrett, Wesley J Tucker, Siddhartha S Angadi, Marc A Adams

**Affiliations:** ^1^ Exercise Science and Health Promotion School of Nutrition and Health Promotion Arizona State University Phoenix, AZ United States; ^2^ College of Nursing and Health Innovation Arizona State University Phoenix, AZ United States

**Keywords:** just in time adaptive interventions, Fitbit, exercise, overweight, inactive, text messaging, SMS, percentile schedule of reinforcement, mHealth

## Abstract

**Background:**

Walking is a widely accepted and frequently targeted health promotion approach to increase physical activity (PA). Interventions to increase PA have produced only small improvements. Stronger and more potent behavioral intervention components are needed to increase time spent in PA, improve cardiometabolic risk markers, and optimize health.

**Objective:**

Our aim is to present the rationale and methods from the WalkIT Trial, a 4-month factorial randomized controlled trial (RCT) in inactive, overweight/obese adults. The main purpose of the study was to evaluate whether intensive adaptive components result in greater improvements to adults’ PA compared to the static intervention components.

**Methods:**

Participants enrolled in a 2x2 factorial RCT and were assigned to one of four semi-automated, text message–based walking interventions. Experimental components included adaptive versus static steps/day goals, and immediate versus delayed reinforcement. Principles of percentile shaping and behavioral economics were used to operationalize experimental components. A Fitbit Zip measured the main outcome: participants’ daily physical activity (steps and cadence) over the 4-month duration of the study. Secondary outcomes included self-reported PA, psychosocial outcomes, aerobic fitness, and cardiorespiratory risk factors assessed pre/post in a laboratory setting. Participants were recruited through email listservs and websites affiliated with the university campus, community businesses and local government, social groups, and social media advertising.

**Results:**

This study has completed data collection as of December 2014, but data cleaning and preliminary analyses are still in progress. We expect to complete analysis of the main outcomes in late 2015 to early 2016.

**Conclusions:**

The Walking Interventions through Texting (WalkIT) Trial will further the understanding of theory-based intervention components to increase the PA of men and women who are healthy, insufficiently active and are overweight or obese. WalkIT is one of the first studies focusing on the individual components of combined goal setting and reward structures in a factorial design to increase walking. The trial is expected to produce results useful to future research interventions and perhaps industry initiatives, primarily focused on mHealth, goal setting, and those looking to promote behavior change through performance-based incentives.

**Trial Registration:**

ClinicalTrials.gov NCT02053259; https://clinicaltrials.gov/ct2/show/NCT02053259 (Archived by WebCite at http://www.webcitation.org/6b65xLvmg).

## Introduction

### Overview

Walking is a low-cost, widely accepted physical activity (PA) associated with significant health benefits [1,2]. However, a meta-analysis by Conn et al [3] found that PA interventions showed improvements of only 14.7 minutes/week (about 2 minutes/day), suggesting more potent interventions are needed. Goal setting is a key component of behavioral theories [4,5] and PA interventions [6]. Though step goals are a key modality to increase PA, differing implementation strategies impede definitive conclusions [7]. Also, inconsistencies in reward structures for goal attainment further impair best practices for behavior change. Principles of operant learning and behavioral economics have the potential to concurrently refine goal setting and incentive strategies in the behavioral sciences [8].

### Percentile Shaping: Adaptive Versus Static Goal Setting

Goal setting approaches are often fixed over time, though typically vary from researcher-assigned [9-12] to participant-selected [7,13,14]. If goals include participant input, it is often to set a starting goal from a baseline value [7]. A review of goal setting approaches for weight loss using PA and diet interventions found mixed results, noting the presence of extensive methodological issues and confounders in most studies [15]. Traditionally, fixed or static goals are the same across individuals (eg, walk 10,000 steps or exercise 30 minutes daily), affording the investigator a simple but insensitive threshold to promote behavior change. A major limitation of static goals is the inherent inflexibility to accommodate the myriad influences on day-to-day behavior (eg, illness, major life events, daily, or other cyclical schedules [16]).

Adaptive goals that adjust frequently and uniquely to an individual’s recent performance may be a more realistic approach to developing flexible yet challenging and attainable goals, but the task remains to standardize treatment dose across participants. Recently, intensively adaptive interventions have gained attention [16,17]. Intensively adaptive interventions require intensive (eg, daily) repeated measures of the tailoring variable(s) and target behavior. Concepts from percentile shaping may be one solution to standardize treatment dose [16,18] and through technology can be coupled with intensive repeated measures to produce intensively adaptive interventions [16,17].

Percentile shaping uses a moving window of recent performance (eg, last 9 observations or days) and a rank-order percentile algorithm to produce adaptive goals that can adjust systematically up or down daily, both within and between individuals, and over time. Percentile shaping capitalizes on the natural variation in behavior to produce personalized goals. Percentile shaping also generates inherently specific, measurable goals that can be explicitly rewarded. Only a handful of studies have tested the use of a percentile shaping approach by providing adapting goals to increase physical activity, and none have orthogonally compared goals derived from percentile schedules with immediate versus delayed reinforcement [16,19,20].

### Reinforcement Schedule: Immediate Versus Delayed

Rewarding small changes in behavior over time is important; however, types and dimensions (eg, latency, schedule) of reinforcement for goal attainment vary widely across interventions [15,16,21-23]. Principles of operant psychology [8] and behavioral economics [24] posit a temporal inconsistency in reward structures for healthy versus unhealthy behaviors. Specifically, “less healthy” behaviors tend to deliver an immediate reward (eg, physical comfort for sedentary behavior), with deleterious effects in the future (eg, lowered physical fitness contributing to heart disease). However, “more healthy” behaviors typically require an immediate cost (eg, physical discomfort for intense exercise) and require a sustained effort to experience delayed rewards (eg, improved physical fitness). As the benefits of healthier behaviors are inherently delayed, the immediate benefit of the less healthy response often wins in the economics of behavior. Interventions that attempt to tip the balance of reward in favor of healthy behaviors through immediate rewards are likely to produce more favorable and longer-term behavior change [16,24-26].

### Purpose and Aims

The purpose is to present the rationale and methods from the Walking Intervention Through Texting (WalkIT) trial—a 4-month, 2x2 factorial randomized controlled trial (RCT) for inactive, overweight, and obese adults. We used a semi-automated text message system to deliver adaptive versus static goals and immediate versus delayed reinforcement. The primary aim was to evaluate whether adaptive goals and immediate reinforcement resulted in a greater change in objectively measured PA compared to the static intervention and delayed reinforcement groups. Daily step counts were measured by a Fitbit device over the course of the 4-month study to evaluate the primary aim. We hypothesized that participants in the adaptive goals and immediate reinforcement groups would increase their average steps/day more than participants in the static goals or delayed reinforcement groups. Secondary aims were to evaluate the effectiveness of the adaptive goal and immediate reinforcement interventions to improve psychological measures, aerobic fitness, and cardiometabolic risk factors.

## Methods

### Overview

The WalkIT trial was a 2x2 factorial RCT conducted over 4 months. Following a 10-day baseline phase to assess usual PA levels measured by Fitbit Zip accelerometers, participants underwent simple randomization using a computerized random number generator for assignment into one of four treatment groups. Main effects of the treatment included Goal Type (adaptive vs static goals) and Reinforcement Type (immediate vs delayed reinforcement). In brief, adaptive goals and immediate reinforcement were based on a percentile-shaping algorithm that adjusted each participant’s goal up and down daily based on their previous nine valid observations (usually the last 9 days) of Fitbit-measured steps. Static goals were set to the recommended 10,000 steps per day and did not change over the course of the study. Participants in the immediate reinforcement group received praise feedback and one reward point each time they met a daily goal, whereas those in the delayed reinforcement group received monthly incentives. All participants received a walking intervention with semi-automated text message–based components. Researchers monitored the text message system for non-standard messages from participants (eg, when a participant asked a question, the research staff was notified), and staff responded through the system.

The Arizona State University Institutional Review Board approved the intervention trial and all the procedures used in data collection. The study is registered as a clinical trial (NCT02053259). See Multimedia Appendix 1 for the CONSORT EHEALTH checklist [27].

### Inclusion and Exclusion Criteria

Participants were generally healthy, inactive, 18-60 years old, with a body mass index (BMI) of 25-55 kg/m^2^ (see Table 1 for complete list of inclusion and exclusion criteria). Initially, criteria were 18-45 years and BMI 25-45 kg/m^2^, but these were increased due to interest and to meet recruitment goals. Attempts were made to contact those previously ineligible who met the expanded criteria. The criteria for determining an inactive participant was defined in two ways. First, physical activity level was screened online with the International Physical Activity Questionnaire (IPAQ) short form. We addressed the limitations of using the IPAQ (eg, recall and social desirability bias) as a pre-screening method by inviting those with ≤1300 metabolic equivalents (MET)-min/week to make an appointment for the office visit. Then, in the 10-day baseline phase prior to randomization, we provided participants with a masked Fitbit (to limit participant reactivity) and monitored wear and steps/day remotely. Inactive participants, defined as those who did not accumulate ≥10,000 steps/day on ≥5 days/week, were randomized to one treatment group. Participants were required to have basic computer literacy, daily access to a personal computer for study-related software, a mobile phone with short message service (SMS, or text) capabilities, and be willing to receive up to 3-5 text messages per day. These criteria were imperative to receiving the mobile health (mHealth) related intervention components.

**Table 1:** Inclusion and exclusion criteria for the WalkIT trial.

Participants		Criteria
**All participants**		
	Home residence	Live in Maricopa County, Arizona.
	Age	Between 18-60 years.
	Body Mass Index	Between 25-55 kg/m^2^.
	Inactive	Not meeting or exceeding physical activity (PA) recommendations (ie, ≥10,000 steps/day on ≥5 days/week).
	Health	No contraindicated condition(s) as assessed via Physical Activity Readiness Questionnaire (PARQ+).
	Medication use	No medication(s) use that prohibits a moderate intensity physical activity program or testing.
	Pregnancy status	Not currently pregnant or planning to become pregnant in the next 4 months.
	Staying within study area	Not planning to leave for ≥10 days or live outside of Maricopa County in the next 4 months.
	Concurrent program	Not currently in a physical activity, diet, or weight loss program (eg, Weight Watchers).
	Computer access	Access to personal Windows or Mac machine on a daily basis.
	Internet access	Access to email and the Internet daily.
	Mobile phone access	Has mobile phone with text messaging; willing to send and receive up to 3-5 texts per day.
**For vascular subset measures**		
	Vaso-active medications	No supplements or over-the-counter medications (eg, calcium, non-steroidal anti-inflammatories) at least 4 days prior to visits.
	Female menstrual phase	Within 7 days of onset of menses or >12 months post-menopause at time of visits.

### Recruitment and Setting

Recruitment emails and paper fliers included a brief study overview, notice of compensation for participating, and instructions on how to receive more information and begin the screening process. Local businesses, government agencies, social networking groups, retail outlets, and university departments were contacted to send the email notice and some elected to post physical fliers for their employees or patrons. Focused recruitment of minority populations was conducted through a free online social group advertisement.

Interested participants were directed to a secure online survey system (Qualtrics, LLC) for a pre-screening step, where they found a brief description of the study and completed the eligibility survey. Those determined to be eligible at pre-screening were contacted via phone and email for a telephone follow-up screening. Written (via online survey check box) and verbal informed consent (via the phone) were obtained at the initiation of each screening, respectively. Over the phone, the study was described in detail to participants, who were then offered opportunities to ask questions about participating and asked to clarify responses from their pre-screening responses to further assess eligibility. Qualified individuals were invited to schedule an appointment at the research office to review the study, provide written informed consent, complete baseline measures, and participate in accelerometer training.

Participants were required to reside in Maricopa County, Arizona, and to agree to make two visits to the research office located in Phoenix, Arizona, for pre- and post-intervention measurements. Rolling recruitment occurred February-August 2014 with data collection completed in December 2014. Weather was anticipated to be an influential confounder as the study occurred chiefly in the warmer months and over a monsoon season. Phoenix has a subtropical dry arid desert climate at low latitude (Köppen climate BWh). Wide variation in seasonal temperatures (eg, average high temperatures: July 41.2°C/106.2°F, December 18.9°C/66.0°F), along with monsoons (which include dust storms and flash floods), may drastically limit outdoor activity on very hot or hazardous days.

The 2013 median annual household income in Maricopa County was US $53,596 [28]. Maricopa County’s majority is white, non-Hispanic (58%), with those identifying as Hispanic or Latino (30%), black (8%), and Asian (4%) being the largest racial/ethnic minority groups [28]. Statewide, 93% of households have access to at least one vehicle [28] and since 2/3 of the state population lives in the recruitment area (ie, Maricopa County), this indicates heavy reliance on car travel within our study area.

### Intervention Groups

**Figure 1:**
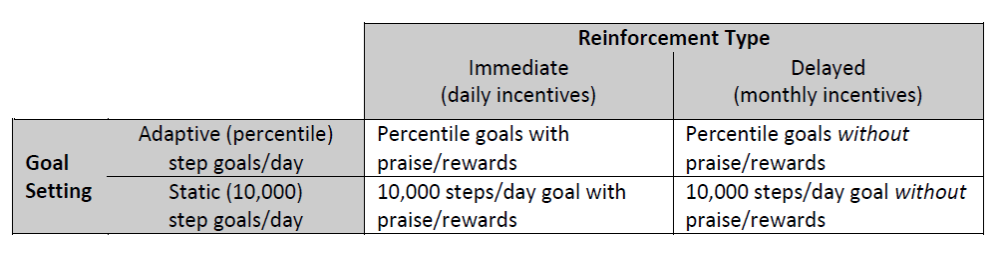
Illustration of 2x2 factorial design.

Figure 1 shows how intervention groups varied by goal type (ie, adaptive vs static goals) and reinforcement type (ie, immediate vs delayed reinforcement) in a 2x2 design. This design allowed all participants to receive a form of the treatment in an effort to reduce attrition and improve compliance over the duration of the study. During the intervention phase, all participants were asked to self-report steps nightly (ie, before midnight) via the study’s interactive text messaging system that acknowledged their report and provided differential feedback based on group status and performance. The factorial design efficiently tested how each component influenced PA without the use of a “no-treatment” control group. All participants were told their ultimate target behavior was 10,000 steps on ≥5 days per week and that daily goals sent via SMS were good for 1 day only.

### Experimental Components

#### Goal Setting

Participants received a goal by text message each day they self-reported their steps. The static intervention groups received the standard 10,000 steps per day goal, with immediate or delayed reinforcement for goal attainment. Participants assigned to the adaptive goal group received performance-based goals based on an algorithm developed by the research team. This algorithm was adapted from recent developments in basic science around percentile schedules of reinforcement [18,29]. The preceding nine observations (typically last 9 days) of data were used to derive a rank-ordered percentile goal. The percentile algorithm requires (1) continuous and repeated measurements of PA, (2) ranking of a sample of behavior (steps/day) from lowest to highest, and (3) calculation of a new goal based on an *n*th percentile criterion.

To illustrate, if a participant’s step count for the preceding 9 days was 1000, 1500, 2600, 4500, 5000, 5700, 6300, 8000, 11,000, rank-ordered from lowest to highest, using a 60th percentile criteria, then 5700 steps becomes the participant’s next goal. The baseline phase provides data for the first goal and then a 9-day “moving window” adapts in each new day’s step count to calculate the next goal. The newest step count observation replaces the oldest step count observation. The 60th percentile was chosen based on previous PA research by Adams [16,20].

It is important to highlight that prescribed adaptive goals always fall within each participant’s recent abilities due to the moving window of the last 9 days. This is distinct from the static intervention group, which receives the commonly recommended goal of at least 8000-10,000 steps 5 days/week, which may be well beyond their current abilities. Because adaptive goals adjust daily, participants were informed that each new goal is good for only one day. We believe this encourages participants to send in step reports daily unprompted.

#### Praise Messages

Several health behavior theories indicate that it is critical to praise improvements to develop new behavior or strengthen a habit [8,30,31]. The combination of goals and feedback was expected to provide a strong PA-shaping program. Delayed reinforcement participants did not receive contingent praise for achieving goals but did receive incentives on a monthly basis. Participants assigned to the immediate reinforcement groups received contingent differential feedback depending on whether a daily goal was met. Participants who did not meet the goal were provided a simple confirmation that steps were entered correctly and provided their next day’s goal (eg, “Steps Received. Goal for 4/1/14 is 4525 steps”). This approach avoids negative messages that could be discouraging. Each time a participant met a goal, a single message from the pool of praise messages was sent to the participant (eg, “Well done! You’re steps closer to good health. Goal for 4/1/14 is 4525”). Some messages used the participant’s first name for enhanced personalization.

#### Reward Points and Incentives

Participants assigned to the delayed reinforcement groups received progressively increasing incentives each month for participating in the study (month 1=$5; months 2 and 3=$10 each; month 4=$20; total $45). Participants assigned to the immediate reinforcement groups received a point-based incentive each time they met a step goal. They had the opportunity to earn a point once per day (110 points possible) when a goal was met by the end of the day. Points were automatically exchanged for incentives ($5 for every 5 points earned) during the study. Participants self-selected their incentive from a list of retail options (eg, Amazon, iTunes, Target) or a charity (ie, the United Way), and all incentives were sent as electronic gift cards. To prevent habituation or satiation, they were allowed to change their choice at any time. Incentive amounts for delayed reinforcement groups approximate the total amount made available to the immediate reinforcement groups to control for cumulative amount of incentives.

### Additional Intervention Components

#### Overview

All four groups received the following: (1) Fitbit Zip, (2) SMS based self-monitoring and reporting of steps per day, (3) brief health information, and (4) text message prompts. Random allocation was performed by a researcher who did not have contact with participants during screening or assessments and who knew them only by participant identification number.

#### Objectively Measured Physical Activity

Participants in all four groups received a commercially available accelerometer (Fitbit Zip, Fitbit Inc.) to wear for the 4-month duration of the study. Participants wore the accelerometer for at least 10 days prior to randomization to an intervention group and continued wearing for the remaining approximately 110 days. The Fitbit clips on clothing near the hip and has a small and unobtrusive form factor, thus accommodating various clothing styles to minimize non-wear. Participants were asked to wear the Fitbit during all waking hours (ie, at least 10 hours) every day for the duration of the study (ie, both the baseline and intervention phases), removing it for sleeping or in circumstances that might submerge it in water (eg, swimming). Fitbit accelerometers have excellent reliability and validity for measuring steps compared to direct observation and Actical accelerometers [32,33]. The Fitbit recorded steps and transmitted the data to the study team via the Internet to verify participants’ texted step reports. The accelerometer’s display was masked during baseline (to avoid participant reactivity) and then unmasked at randomization. Participants were also asked to sync their Fitbit each day using a personal computer connected to the Internet and the Fitbit sync dongle. Instructions on how to install the software were provided and demonstrated in the lab. The study team initiated and created study-managed accounts on the Fitbit website so participants were *not* able to access or view activity history, nutrition trackers, “badges” earned, social media interfaces, and other online tools that could have acted as potential confounders. To further limit access to the Fitbit website, we did not allow participants to sync their Fitbit using Fitbit’s mobile phone app because this required logging into the study-managed account. Fitbit, Inc. allowed researcher access to their Application Programming Interface (API) but otherwise was not involved with this project.

#### Text Message System

The study’s software engineer developed a proprietary automated text message system with the principal investigator. The texting system was the “front end” for participants to interact with the study and used a commercial SMS gateway service (Twilio) with a designated study SMS phone number. Participants in all four groups were instructed to send a “step report” text message to this number each night after 8 p.m. The “step report” is a daily step count in a specified format (eg, “5555 today”). The system was fully automated to recognize step reports, in a pre-determined set of natural language patterns, from all other types of messages. All SMS traffic was logged in a server database. Automated feedback was provided as per the participant’s intervention assignment when a step report was obtained. Participants in the immediate reinforcement groups received a US $5 incentive email automatically from the system upon meeting a daily goal when the 5^th^ point was earned.

**Figure 2:**
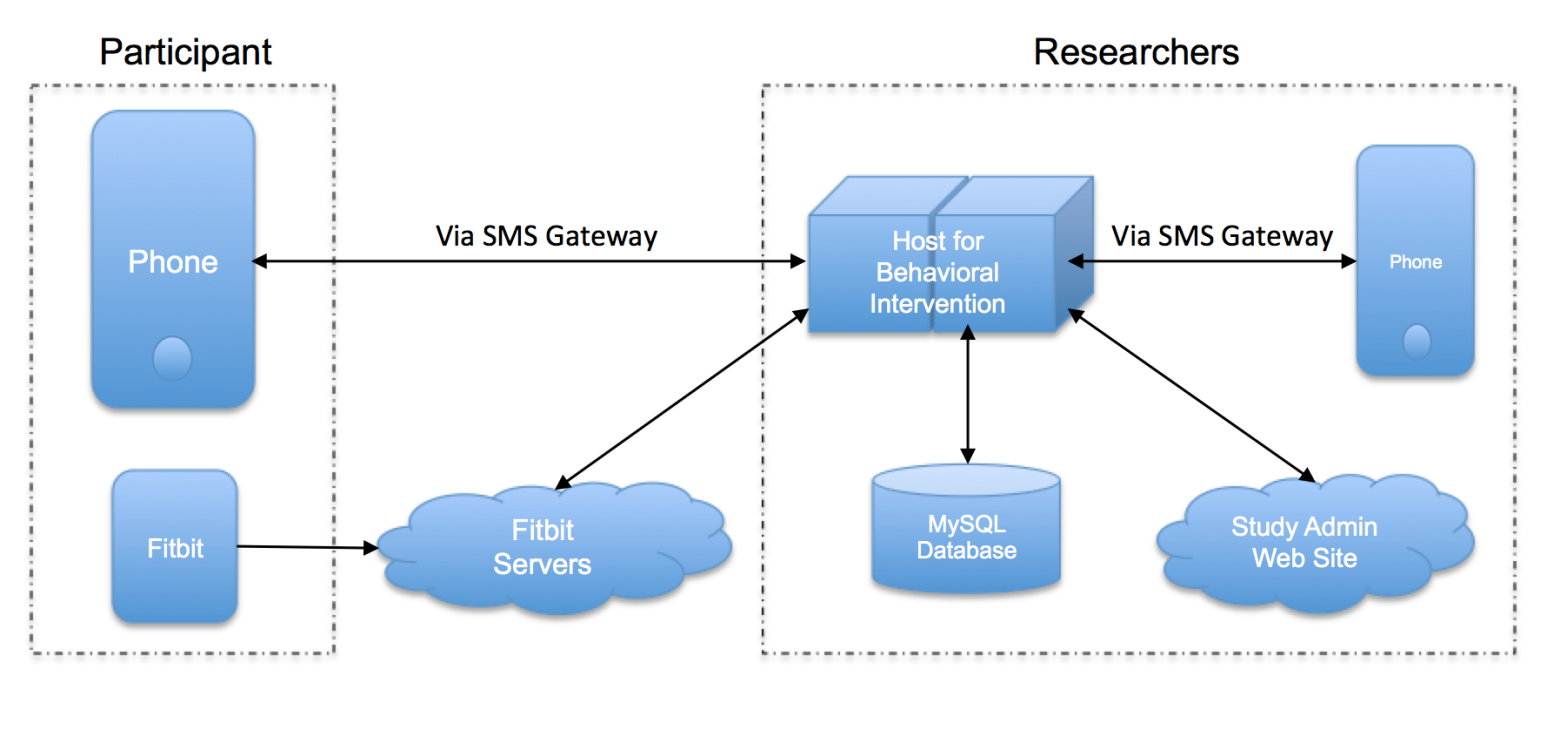
Schematic for intensive adaptive intervention system.

Text messages were sent to all participants daily through this same study SMS number. Participants could text message “goal” at any time to receive an automated reminder of their step goal for that day. When a message was not recognized by the system (eg, “I lost my Fitbit”), it was immediately forwarded to the on-duty researcher’s mobile device with a prefix of the participant’s study identification number. The system facilitated researcher-initiated messages to participants through the system’s phone number (ie, all messages appeared to come from the SMS phone number regardless of the mobile device it originated from). Researchers could send texts to a specific participant or to all participants as group (see Figure 2 for a diagram of the system).

#### Health Information Brochures

Upon randomization, participants in all four groups were sent two brochures on PA via email. A US Health and Human Services brochure [34] presents information on overcoming barriers to being active, initiating a PA routine, and recommendations on quantity of PA to evoke health benefits. An America on the Move Foundation brochure [35] suggests 100 ways to increase steps (eg, “take your dog for a walk”) throughout the day. Participants in all groups received the same materials on the first intervention day only.

#### Text Message Prompts

All participants in the intervention phase received daily text message prompts (≤160 characters) to encourage PA, except when Ecological Momentary Assessment (EMA) questions were administered (see Table 2). A randomly selected message (or EMA question) was sent at a random time of day between the hours of 8 a.m. and 6 p.m. local time. The research team developed a pool of messages that complemented or expanded on the health information brochures. The prompts included motivational quotes, health risks of inactivity, benefits of PA, and encouragement to be active (eg, “It doesn’t matter how old you are – it’s never too early or too late to become physically active so start today; only then will you start to see results!”). Acknowledging that maintaining participation over a 4-month study is often difficult, these unvalidated prompts were primarily a mechanism to remind participants of their involvement and served as somewhat of a disguise for the experimental components under manipulation (ie, the goal-setting and reinforcement types).

### Office Visits and Secondary Outcome Measures

Eligible participants visited the laboratory twice for about 2 hours each time. The initial visit included the written informed consent, physical activity PAR-Q+, the pre-measures as listed in Table 2, and training on the Fitbit and the texting system. At the second visit, the participant returned the Fitbit, completed post-measures (similar to pre-, except where noted in Table 2 [36-49]), and was debriefed regarding the study purpose. Data collection staff were blinded to treatment allocation at pre- and post-measures. However, it was impossible to blind participants as treatment groups were explained in the consent process.

**Table 2:** Secondary outcome measures.

Measure		Description	Frequency		
			Pre-post	Once	Other
**Self-administered computerized surveys**					
	IPAQ long form	IPAQ long form; 31-item survey designed to capture details on domain-specific physical activity with acceptable test-retest (*r*=.8) and criterion validity (*r*=.3) [36] and intraclass correlation coefficient=.27-.71 [37].	x		
	Neighborhood	Neighborhood Environment Walkability Scale abbreviated; 54-item survey to measure neighborhood characteristics [38-40].		x^b^	
	Monetary choice	Delayed discounting protocol using 27-item self-administered questionnaire [41,42].		x^c^	
	Satisfaction	Consumer satisfaction style questionnaire for rating experience and providing feedback; question number and type differed by intervention assignment.		x^c^	
	Self-Efficacy	Single-item Ecological Momentary Assessment (EMA) of self-efficacy (0-9 Likert-type scale) delivered via SMS on 21 random intervention days. Item language based on previous work [43,44].			x
**Researcher performed laboratory measures**					
	Height and weight	Measured using digital stadiometer and scale (Seca 284 measuring station, Seca GmbH & co. KG).	x		
	Aerobic fitness	Aerobic capacity (VO_2peak_) estimated using a submaximal continuous treadmill ramp protocol (modified Balke) and the Foster equation [45-47].	x		
	Body composition^a^	Dual-energy x-ray absorptiometry (Lunar iDXA, GE Healthcare).	x		
	Blood pressure^a^	Brachial and central blood pressure assessed during pulse wave analysis using Sphymocor XCEL (AtCor Medical Inc) [48,49].	x		
	Arterial stiffness^a^	Carotid-femoral pulse wave velocity assessed using Sphymocor XCEL (AtCor Medical Inc) [48,49].	x		
	Biochemical^a^	Venous blood samples for cardiovascular risk and inflammatory markers; post-centrifugation samples archived in aliquots at -80°C.	x		

^a^Measured in the vascular subset of participants.

^b^Measured once at initial visit; second done at follow-up only if moved during study.

^c^Measured at follow-up visit only.

### Statistical Analysis

#### Power and Sample Size Determination

To estimate the sample size required to test the main aim of changes to steps/day, we conducted a set of simulations using a SAS macro developed by Psioda [50] and effect size estimates derived from findings reported by Adams et al [16]. Across the simulation runs, we varied the number of participants (Ns of 60-96), the magnitude of change from baseline to intervention Phase (1200 vs 1600 steps/day), magnitude of Goal Type (Static vs Adaptive) x Phase interaction effect (800 vs 1200 steps/day between-group difference in the magnitude of the Phase effect; compare to 1130 steps/day difference reported by Adams et al [16]), magnitude of Reinforcement Schedule (Delayed vs Immediate) x Phase interaction (300 vs 500 steps/day between-group difference in the magnitude of the Phase effect), and the Goal x Reinforcement x Phase interaction (150 vs 300 steps/day difference in Phase effect over and above the main effects and 2-way interaction effects). The simulations revealed that under the most conservative sets of assumptions (ie, sets comprising combinations of the smallest effect magnitudes) and an assumed attrition rate of 20%, a total initial sample size of N=100 participants (n=25 per group) would be required to have power of .80 or greater to detect hypothesized interaction effects.

#### Data Analysis

We plan to first examine univariate and bivariate statistics to evaluate distributional properties of outcome measures and to identify potentially relevant confounders and covariates. We will also evaluate psychometric properties (eg, internal consistency) of self-report multi-item measures of psychosocial variables. Where warranted, we will apply transformations (eg, natural log) to normalize distributions of outcome measures. We will examine main effects of and interactions among Phase (Baseline vs Intervention), Goal Type (Static vs Adaptive), and Reinforcement Type (Delayed vs Immediate) using a generalized linear mixed (ie, random effects or multilevel regression) modeling approach, with repeated assessments of PA (ie, both steps/day and minutes above various step/min cadence levels) treated as nested within persons. To minimize collinearity among interaction terms and constituent linear effects, we will use effect-coded indicators (ie, -1/1) as opposed to dummy coded (ie, 0/1) indicators for dichotomous predictors. In all models, we will account for (1) effects of covariates identified in preliminary analyses, (2) linear, quadratic, and cyclical (weekly, monthly) time effects, (3) random variation in person-level intercepts, and (4) autocorrelation among residuals. All analyses will be conducted using mixed modeling procedures in SAS 9.4 (eg, PROC MIXED, PROC GLIMMIX) and R (eg, lme4).

We will model the main effect of each intervention component (either Goal Type or Reinforcement Type) on changes in PA (steps/day and cadence) from baseline to 4 months via Intervention x Phase (Baseline vs Intervention) interactions. The interaction between interventions will be examined via a Goal Type x Reinforcement Type x Phase interaction effect, with planned contrasts comparing PA change in the Adaptive Goal + Immediate Reinforcement condition to PA change in the other groups. Secondary analyses will be dependent on the specific research question and the most appropriate statistical methods for the design.

#### Missing Data

Given the potential for non-ignorable missingness in our outcome data, we will explore various strategies for mitigating potential biases in estimates and loss of statistical power due to missing data, including standard intent-to-treat approaches, full information maximum likelihood-estimated models, and analysis of multiply-imputed datasets, to be followed by sensitivity analyses assessing robustness of conclusions drawn from each approach.

## Results

This study completed data collection in December 2014, but data cleaning and preliminary analyses are still in progress. We expect to complete analysis of the main outcomes in late 2015 to early 2016.

## Discussion

### Principal Considerations

This study integrates measures of behavior change and physiological outcomes to evaluate intervention strategies and mechanisms that improve health through adoption of walking behaviors over 4 months in an inactive, overweight/obese adult sample. The study examines the effects of two experimental factors: (1) percentile shaping to produce performance-based adaptive goals, versus typical static goals of 10,000 steps/day, and (2) reward structure using principles of behavioral economics (ie, US $1 per daily goal achieved, obtained immediately as goal achievement is reported), versus a delayed incentive. The group with a combination of static goals and delayed reinforcement approximates procedures found in practical settings (eg, a physician offering a PA brochure, recommending 10,000 steps/day, and giving a pedometer to a patient) with the difference being a predetermined monthly reward for continuing with the study—a common approach in many research studies [16,20,51,52].

Our factorial study design allows examination of the independent and joint effects of these components and explores the promise of percentile shaping and small immediate rewards to optimize behavioral interventions. Our work will contribute to the field by testing specific methodologies that link behavior change theory to practical applications. The limited body of research on shaping to improve PA shows complementary results, even with differing methodological approaches [16,19,20]. Further, employing mHealth strategies, such as SMS for treatment delivery and reward mechanisms, and wearable activity monitoring with feedback and wireless upload, enhance the overall treatment and theoretical fidelity [53] while capitalizing on the omnipresence of mobile technology.

### Limitations

Potential limitations of this study include limited generalizability due to convenience sampling, although random allocation to the treatment group improves internal validity and reduces selection bias. Inclusion criteria may also limit generalizability as only generally healthy persons with a BMI classification as overweight or obese were included. Further limitations include a 4-month intervention length, which may not be long enough for some individuals to fully adopt successful walking routines. Also, without a post-intervention period follow-up, we will not be able to determine behavioral maintenance.

Strengths include the relatively large sample size, especially considering the extensive laboratory visits (approximately 2 hours each). The intensive repeated measures design is important for monitoring PA behavior to provide continual performance-based feedback via percentile shaping. We also included a large number of pre-menopausal women in the physiological measures, which is important due to underrepresentation in studies that limit inclusion to men and post-menopausal women when assessments involve biomarkers such as biochemical assays and arterial stiffness. Increasing time spent in PA is independently beneficial to health [54] especially for inactive populations and regardless of weight status [55-57]. We aim to better elucidate the link between behavior change and mediators of physiological and cardiometabolic health markers.

### Conclusion

The Walking Interventions Through Texting (WalkIT) trial will further the understanding of theory-based intervention components to increase the PA of men and women who are healthy, insufficiently active, and are overweight or obese. With the overwhelming number of options interventionists have to use in health promotion, it is useful to look mechanistically at specific intervention components to optimize the treatment with economical, scalable mHealth methods. Though many studies have investigated walking interventions through a variety of methods, WalkIT is among the first directing the focus to the individual components of combined goal setting and reward structures in a factorial design to increase walking. The WalkIT trial is expected to produce results useful to future research interventions and perhaps industry initiatives, primarily focused on mHealth, goal setting, and those looking to promote behavior change through performance-based incentives.

## Appendix

Multimedia Appendix 1 CONSORT-EHEALTH checklist V1.6.2 [27].

## Abbreviations

API: Application Programming Interface
EMA: Ecological Momentary Assessment
IPAQ: International Physical Activity Questionnaire
MET: metabolic equivalents
PA: physical activity
PARQ+: Physical Activity Readiness Questionnaire
RCT: randomized control trial
SMS: short message service
WalkIT: Walking Interventions Through Texting
