# Supplementary material for: The Walking Interventions Through Texting (WalkIT) Trial: Rationale, Design, and Protocol for a Factorial Randomized Controlled Trial of Adaptive Interventions for Overweight and Obese, Inactive Adults
Source: JMIR Res Protoc. 2015 Sep 11;4(3):e108. doi: 10.2196/resprot.4856 (PMC4704955; doi:10.2196/resprot.4856)
Supplement: Multimedia Appendix 1 [file resprot_v4i3e108_app1.pdf]

CONSORT-EHEALTH Checklist V1.6.2 Report

(based on CONSORT-EHEALTH V1.6), available at [http://tinyurl.com/consort-ehealth-v1-6].

4856

Date completed

6/24/2015 0:53:29

by

Jane

Walking Interventions through Texting (WalkIT) Trial: Rational, design and protocol for a factorial randomized controlled trial of adaptive interventions for inactive adults

TITLE

1a-i) Identify the mode of delivery in the title

Yes, "Interventions through Texting" in the title indicates that text messaging is the intervention delivery method.

1a-ii) Non-web-based components or important co-interventions in title

Yes, "adaptive interventions" in the title indicates an important co-intervention.

1a-iii) Primary condition or target group in the title

Yes, "for inactive adults" in the title indicates the target group.

ABSTRACT

1b-i) Key features/functionalities/components of the intervention and comparator in the METHODS section of the ABSTRACT

Yes, "2 x 2 factorial RCT" and "Experimental components included adaptive vs. static steps/day goals, and immediate vs. delayed rewards." in the methods section of the abstract explain the comparator groups.

1b-ii) Level of human involvement in the METHODS section of the ABSTRACT

Yes, "semi-automated, text message (SMS) based walking interventions" in the methods section of the abstract describe the level of human involvement.

1b-iii) Open vs. closed, web-based (self-assessment) vs. face-to-face assessments in the METHODS section of the ABSTRACT

Yes, "Participants were recruited through email listservs and websites affiliated with the university campus, community businesses and local government, social groups and social media advertising. " in the methods section of the abstract describe the recruitment methods.

Yes, "Secondary outcomes...measures performed pre/post in a laboratory setting" in the methods section of the abstract explain the face-to-face components.

1b-iv) RESULTS section in abstract must contain use data

No, as this manuscript is focusing on the rationale, methods and study protocol, this is not being presented here.

1b-v) CONCLUSIONS/DISCUSSION in abstract for negative trials

Yes, there is a discussion section, but negative results have not been found as this paper is a protocol manuscript that is not reporting results.

INTRODUCTION

2a-i) Problem and the type of system/solution

Yes, the chief problem is the lack of strong intervention techniques to increase physical activity. Early in the introduction we cite, "A meta-analysis by Conn et al. found PA interventions showed improvements of only 14.7 min/week (about 2 min/day), suggesting more potent interventions are needed [3]."

Also, specific problems to be addresses goal setting and rewards for meeting goals, and we state, "Though step goals are a key modality to increase PA, differing implementation strategies impede definitive conclusions [7]. Also, inconsistencies in rewards for goal attainment further impair best practices for behavior change."

The specific population is, "inactive, overweight adults" and the type of system/solutions that is the object of the study is, "a semi-automated text message system to deliver the adaptive vs. static goals and immediate vs. delayed rewards." In our view, this system can be either stand alone or incorporated into a broader health care program, so this is not explicitly discussed in the manuscript.

2a-ii) Scientific background, rationale: What is known about the (type of) system

Yes, the scientific background is discussed, "Principles of operant learning and behavioral economics can concurrently refine goal setting and incentive strategies in the behavioral sciences." with a dedicated paragraph to discuss both percentile shaping (operant learning) and reinforcement schedules (behavioral economics). In these introductory paragraphs, we summarize and cite the previous work which motivates this study.

As for the choice of the comparator, we briefly discuss the design as a "2 x 2 factorial randomized controlled trial" and that the experimental components are, "the adaptive vs. static goals and immediate vs. delayed rewards" which is discussed at greater length in the methods section.

METHODS

3a) CONSORT: Description of trial design (such as parallel, factorial) including allocation ratio

Yes, we specify objectives and hypothesis in the introduction, "The primary aim will evaluate whether the adaptive interventions result in a greater change in PA ... compared to the static intervention groups. We hypothesized that participants in the adaptive goals and incentive groups would increase their average steps/day... Secondary aims will evaluate participants' satisfaction ... anthropometric, psychological, cardiovascular fitness, and cardiometabolic risk factors."

3b) CONSORT: Important changes to methods after trial commencement (such as eligibility criteria), with reasons

Yes, the only important change after trial commencement was eligibility criteria, "Participants were generally healthy, insufficiently active, 18 to 60 years old, with a BMI 25 - 55 kg/m2... The initial two months of recruitment (of six total months), criteria was 18 - 45 years and BMI 25 – 45 kg/m2, but criteria was increased to meet recruitment goals and due to interest from this population. Those previously ineligible, but then met the new criteria were contacted after Review Board approval of protocol modification."

3b-i) Bug fixes, Downtimes, Content Changes

No, we do not have major bug fixes, downtimes, content changes or unexpected events to the our system report.

4a) CONSORT: Eligibility criteria for participants

Yes, we specify eligibility criteria as, "Participants were generally healthy, insufficiently active, 18 to 60 years old, with a BMI 25 - 55 kg/m2; see Table 1 for complete list of inclusion and exclusion criteria."

We discuss recruitment strategies in two paragraphs within the manuscript, "Recruitment emails and paper fliers included a brief study overview, notice of compensation for participating, and instructions on how to receive more information and begin the screening process."

Also, we explain that, "Interested participants were directed to a secure online survey system... for a pre-screening..." which occurred prior to phone screening and initial visit to the laboratory.

4a-i) Computer / Internet literacy

Yes, we state that, "Participants were required to have basic computer literacy, daily access to a personal computer for study-related software, a mobile phone with short message system (text or SMS) capabilities, and be willing to receive up to 3-5 text messages per day."

4a-ii) Open vs. closed, web-based vs. face-to-face assessments:

Yes, we explain how recruitment occurred as addressed in item 4a as well as expanding on that by including, "Local businesses, government agencies, social networking groups, retail outlets, and university departments were contacted... Focused recruitment of minority populations occurred through a free online social group advertisement."

We also explain the face-to-face components, "...eligible participants visited the study laboratory twice, for approximately two hours each time."

4a-iii) Information giving during recruitment

Yes, and we addressed much of this in item 4a above. We also add, "Written, via online survey, and verbal informed consent were obtained at the initiation of both screenings. Over the phone, the study was described in detail to participants, who were then offered opportunities to ask questions about participating and asked to clarify responses from their pre-screening responses to further assess eligibility."

4b) CONSORT: Settings and locations where the data were collected

Yes, the setting of the study are thoroughly described, some of which is addressed in item 4a, and 4a-ii above.

Location, socioeconomics, cultural environment, and climate are described thoroughly, "Participants were required to reside in Maricopa County, Arizona, USA... Phoenix has a subtropical dry arid desert climate at low latitude (Köppen climate BWh) with wide variation in daily temperatures across seasons... Maricopa County in 2013...median annual household income of US$ 53,596[24]. Statewide, 93% of households have access to at least one vehicle [24], indicating reliance on car travel."

4b-i) Report if outcomes were (self-)assessed through online questionnaires

Yes, outcomes are clearly reported as objectively measured, "...in all four groups received a commercially available accelerometer...to wear for the 4-month duration of the study."

Secondary measures were either administered surveys or researcher performed laboratory measures, "eligible participants visited the study laboratory twice, for approximately two hours ...included the written informed consent, PAR-Q+ health screening, pre-intervention measures... "

Also, Table 2 specifies the surveys and laboratory measures conducted.

4b-ii) Report how institutional affiliations are displayed

We do not describe this in the manuscript. As it was integral to the design for participants to visit our laboratory, we had to include institutional affiliation and do not think it will bias results.

5) CONSORT: Describe the interventions for each group with sufficient details to allow replication, including how and when they were actually administered

5-i) Mention names, credential, affiliations of the developers, sponsors, and owners

Yes, we describe our developer affiliation (in-house) and the technical services used for the intervention, "The study's software engineer developed a proprietary automated text message system...and used a commercial SMS gateway service (www.twilio.com/sms) with designated study SMS phone number."

Also, we state, "Fitbit, Inc. allowed access to their Application Programing Interface (API), but otherwise was not involved with this project."

5-ii) Describe the history/development process

Yes, we describe the development of the intervention, "This algorithm was adapted from recent developments in basic science around schedules of reinforcement [25]."

And specifically, "The preceding nine observations...were used to derive a rank-ordered percentile goal...The 60th percentile was used based on previous PA research by Adams [17, 26]."

5-iii) Revisions and updating

No, we do not have major revisions, updating, or content changes to the intervention or to the our system to report.

5-iv) Quality assurance methods

Yes, we used a validated commercially available accelerometer to collect the main outcome data of steps/day, "Fitbit accelerometers have excellent reliability and validity for measuring steps compared to direct observation and Actical accelerometers [29, 30]. The Fitbit recorded steps and transmitted the data to the study team via the Internet. "

5-v) Ensure replicability by publishing the source code, and/or providing screenshots/screen-capture video, and/or providing flowcharts of the algorithms used

Yes, the intervention was delivered via a semi-automated SMS system, which is diagrammed in the manuscript, "Text message prompts were sent to all participants daily through this same study SMS number... Participants could text message "goal" at any time to receive an automated reminder of their step goal..When a message not recognized by the SMS system (e.g., "I lost my Fitbit"), it was immediately forwarded to the on-duty researcher's mobile device...see Figure 2 for illustration of SMS system."

5-vi) Digital preservation

No, thought this could be important to other eHealth projects, we believe this is not relevant here. Our intervention was delivered via SMS and participants accessed it through their personal mobile device.

5-vii) Access

No, though likely relevant in other papers, we believe this is not relevant here. Our intervention was primarily delivered via SMS and participants accessed it through their personal mobile device.

5-viii) Mode of delivery, features/functionalities/components of the intervention and comparator, and the theoretical framework

Yes, and as requested in item 2a-ii, theoretical framework used to design experimental components is discussed in this manuscript.

Other features are also discussed, such as text message prompt content with example, "prompts included motivational quotes, health risks of inactivity, benefits of PA, and encouragement to be active (e.g. "It doesn't matter how old you are – it's never too early or too late to become physically active so start today; only then will you start to see results!")." Also, there are two paragraphs describing the SMS system and has an accompanying diagram, Figure 2.

5-ix) Describe use parameters

Yes, we describe instructions to participants for use of the accelerometer to, "...wear the accelerometer during all waking hours (i.e., at least 10 hours) every day for the duration of the study..." and that, "All participants were told their ultimate target behavior was 10,000 steps on ≥ 5 days per week." Frequency of use was describe as, "During the intervention phase all participants were asked to self-report steps nightly via the study's interactive text messaging..."

5-x) Clarify the level of human involvement

Yes, as mentioned in items 1b-ii, 2a-i, 4b-i and 5-v, there was human involvement to a limited extent. We refer the reader to those sections for direct quotes, but will reiterate that the SMS system was "semi-automated" with human observers being notified when non-standard texts were received, and that pre-post laboratory measures were collected via research staff.

5-xi) Report any prompts/reminders used

Yes, a paragraph is dedicated in the manuscript describing the prompt messages, briefly, "All participants in the intervention phase received daily SMS text message prompts (≤160 characters) to encourage [physical activity]..."

Also, as described in item 5-v above, participants could retrieve their daily step goal via the SMS system anytime.

5-xii) Describe any co-interventions (incl. training/support)

Although potentially important in other investigations, no co-interventions took place during this study.

6a) CONSORT: Completely defined pre-specified primary and secondary outcome measures, including how and when they were assessed

Yes, we describe both primary and secondary outcome measures thoroughly. Steps/day is the primary outcome, and there is a lengthy paragraph describing the accelerometer used to capture this data. Briefly, "Participants in all four groups received a commercially available accelerometer (Fitbit ZipTM, Fitbit Inc., San Francisco, CA, USA) to wear for the 4-month duration of the study."

Secondary measures also have a dedicated paragraph and Table 2 describing their how and when assessment occurred.

6a-i) Online questionnaires: describe if they were validated for online use and apply CHERRIES items to describe how the questionnaires were designed/deployed

This may be important for other studies, but our computerized questionnaires were administered in the research lab under a supervised setting.

6a-ii) Describe whether and how "use" (including intensity of use/dosage) was defined/measured/monitored

This may be relevant in other studies, but we are not presenting here as the focus of this manuscript is to describe methods.

6a-iii) Describe whether, how, and when qualitative feedback from participants was obtained

Yes, qualitative feedback was obtained and described in the manuscript Table 2, "Satisfaction: Consumer satisfaction style questionnaire for rating experience and providing feedback for the specific intervention group assigned."

6b) CONSORT: Any changes to trial outcomes after the trial commenced, with reasons

Yes, but the important change after trial commencement was eligibility criteria as reported in item 3b, above.

7a) CONSORT: How sample size was determined

7a-i) Describe whether and how expected attrition was taken into account when calculating the sample size

Yes, we dedicate a paragraph to power and sample size calculations, in which we state, "...simulations revealed that under the most conservative sets of assumptions...a total sample size N = 100 participants (n = 25 per group) would be required to have power of .80...This sample size assumes an attrition rate of 25%."

7b) CONSORT: When applicable, explanation of any interim analyses and stopping guidelines

No, we did not conduct interim analyses or set stopping guidelines as the intervention focused on walking and required intensively repeated measures. Also, the time-frame was did not permit such analyses as it was relatively short at 10 months (February - December 2014), especially considering the recruitment period was 6 months and the intervention period was 4 months for each participant.

8a) CONSORT: Method used to generate the random allocation sequence

Yes, we state, "...participants underwent simple randomization using a computerized random number generator..."

8b) CONSORT: Type of randomisation; details of any restriction (such as blocking and block size)

Yes, as noted in item 8a, simple randomization was use.

9) CONSORT: Mechanism used to implement the random allocation sequence (such as sequentially numbered containers), describing any steps taken to conceal the sequence until interventions were assigned

Yes, as noted in item 8a, a computer random number generator was use.

10) CONSORT: Who generated the random allocation sequence, who enrolled participants, and who assigned participants to interventions

Yes, we safeguarded random allocation sequence, "Random allocation was performed by a researcher who did not have contact with participants during screening or assessments and who knew them only by participant ID number."

11a) CONSORT: Blinding - If done, who was blinded after assignment to interventions (for example, participants, care providers, those assessing outcomes) and how

11a-i) Specify who was blinded, and who wasn't

Yes, outcome assessors were blinded, but participants (necessarily) were not, "Data collection staff were blinded to treatment allocation at pre- and post-intervention measures, however it was impossible to blind participants."

11a-ii) Discuss e.g., whether participants knew which intervention was the "intervention of interest" and which one was the "comparator"

Yes, as all interventions received a form of the treatment, none were presented as a control group comparator, stated in the manuscript as, "This design allowed all participants to receive a form of the treatment in an effort to reduce attrition and improve compliance over the duration of the study."

11b) CONSORT: If relevant, description of the similarity of interventions

Yes, we discuss the similarity and differences of interventions in several paragraphs under the subheading "Intervention Groups" which, due to length, we will not include here.

12a) CONSORT: Statistical methods used to compare groups for primary and secondary outcomes

Yes, we devote multiple paragraphs to statistical methods including sub-subheadings for "power and sample size" and "data analysis" for primary outcomes. As secondary analyses are yet to be determines, we state, "Secondary analyses will be dependent on the specific research question and the most appropriate statistical methods for the design."

12a-i) Imputation techniques to deal with attrition / missing values

Yes, we describe this in the "missing data" section, "Given the potential for nonignorable missingness in our outcome data, we will explore various strategies for mitigating potential biases in estimates and loss of statistical power due to missing data..."

12b) CONSORT: Methods for additional analyses, such as subgroup analyses and adjusted analyses

Yes, although as indicated in item 12a, secondary analyses are only mentioned briefly.

RESULTS

13a) CONSORT: For each group, the numbers of participants who were randomly assigned, received intended treatment, and were analysed for the primary outcome

Not applicable to this methods and protocol manuscript.

13b) CONSORT: For each group, losses and exclusions after randomisation, together with reasons

Not applicable to this methods and protocol manuscript.

13b-i) Attrition diagram

Not applicable to this methods and protocol manuscript.

14a) CONSORT: Dates defining the periods of recruitment and follow-up

Yes, we state, "Rolling recruitment occurred between February and August 2014."

14a-i) Indicate if critical "secular events" fell into the study period

No critical secular events occurred.

14b) CONSORT: Why the trial ended or was stopped (early)

The trial was not stopped early.

15) CONSORT: A table showing baseline demographic and clinical characteristics for each group

Not applicable to this methods and protocol manuscript.

15-i) Report demographics associated with digital divide issues

Not applicable to this methods and protocol manuscript.

16a) CONSORT: For each group, number of participants (denominator) included in each analysis and whether the analysis was by original assigned groups

16-i) Report multiple "denominators" and provide definitions

Not applicable to this methods and protocol manuscript.

16-ii) Primary analysis should be intent-to-treat

Not applicable to this methods and protocol manuscript.

17a) CONSORT: For each primary and secondary outcome, results for each group, and the estimated effect size and its precision (such as 95% confidence interval)

Not applicable to this methods and protocol manuscript.

17a-i) Presentation of process outcomes such as metrics of use and intensity of use

Not applicable to this methods and protocol manuscript.

17b) CONSORT: For binary outcomes, presentation of both absolute and relative effect sizes is recommended

Not applicable to this methods and protocol manuscript.

18) CONSORT: Results of any other analyses performed, including subgroup analyses and adjusted analyses, distinguishing pre-specified from exploratory

Not applicable to this methods and protocol manuscript.

18-i) Subgroup analysis of comparing only users

Not applicable to this methods and protocol manuscript.

19) CONSORT: All important harms or unintended effects in each group

Not applicable to this methods and protocol manuscript.

19-i) Include privacy breaches, technical problems

We did not have privacy breaches or technical problems, and have not evaluated data for yet for unintended effects.

19-ii) Include qualitative feedback from participants or observations from staff/researchers

Not applicable to this methods and protocol manuscript.

DISCUSSION

20) CONSORT: Trial limitations, addressing sources of potential bias, imprecision, multiplicity of analyses

20-i) Typical limitations in ehealth trials

Yes, we state foreseeable limitations in a dedicated paragraph in the discussion section.

21) CONSORT: Generalisability (external validity, applicability) of the trial findings

21-i) Generalizability to other populations

Yes, we briefly describe limits to generalizability in the limitations paragraph of the discussion.

21-ii) Discuss if there were elements in the RCT that would be different in a routine application setting

Not applicable to this methods and protocol manuscript.

22) CONSORT: Interpretation consistent with results, balancing benefits and harms, and considering other relevant evidence

22-i) Restate study questions and summarize the answers suggested by the data, starting with primary outcomes and process outcomes (use)

We do included a brief discussion section and restate the research framework, but do not summarize the data as that is not applicable to this methods and protocol manuscript.

22-ii) Highlight unanswered new questions, suggest future research

Not applicable to this methods and protocol manuscript.

Other information

23) CONSORT: Registration number and name of trial registry

Yes, "Trial Registration: ClinicalTrials.gov ID: NCT02053259."

24) CONSORT: Where the full trial protocol can be accessed, if available

This manuscript is meant to be the full trial protocol.

25) CONSORT: Sources of funding and other support (such as supply of drugs), role of funders

This was not a sponsored project.

X26-i) Comment on ethics committee approval

Yes, we state, "The University Institutional Review Board approved the intervention trial and all the procedures used in data collection. The study is registered as a clinical trial (ClinicalTrials.gov ID: NCT02053259)."

x26-ii) Outline informed consent procedures

Yes, we state "Written, via online survey check box, and verbal informed consent were obtained at the initiation of both screenings." During laboratory visits participants did, "provide written informed consent, complete baseline measures, and participate in accelerometer training."

X26-iii) Safety and security procedures

Yes, we assigned participant ID numbers and used university-licensed secured online systems to record sensitive participant information.

X27-i) State the relation of the study team towards the system being evaluated

This was not a sponsored project and all parties involved were university affiliated researchers.
